# Prognostic impact of perioperative blood transfusions on oncological outcomes of patients with bladder cancer undergoing radical cystectomy: A systematic review

**DOI:** 10.1080/2090598X.2020.1859055

**Published:** 2020-12-10

**Authors:** Yannic Volz, Lennert Eismann, Paulo L. Pfitzinger, Jan-Friedrich Jokisch, Alexander Buchner, Boris Schlenker, Christian G. Stief, Gerald B. Schulz

**Affiliations:** Department of Urology, Ludwig-Maximilians University, Munich, Germany

**Keywords:** Bladder cancer, cystectomy, oncology, blood transfusion, urothelial carcinoma, prognostic marker

## Abstract

**Objective**: To conduct a systematic review of whether blood transfusions may be associated with worse outcomes for patients with bladder cancer treated with radical cystectomy (RC), as there has been a recent increase in studies addressing this clinically relevant topic.

**Methods**: PubMed, Ovid Medical Literature Analysis and Retrieval System Online (MEDLINE), Google Scholar, and the ClinicalTrials.gov databases were searched with pre-specified search terms for studies published between January 2010 and May 2020. The systemic review was conducted according to the Preferred Reporting Items for Systematic Reviews and Meta-Analyses (PRISMA) guidelines.

**Results**: A total of 17 studies with 19 627 patients were included after 183 records were screened for eligibility. In all, 10 studies proposed perioperative blood transfusion to be associated with impaired prognosis regarding overall survival, nine studies regarding cancer-specific and four studies regarding recurrence-free survival. The timing of blood transfusion might affect patient outcomes. Notably, several studies did not find a significant correlation between blood transfusions and prognosis. As all studies to date are of retrospective design, the grade of evidence is still limited.

**Conclusions**: Despite the lack of prospective trials, perioperative blood transfusion may lead to worse oncological outcomes. These results, as well as known non-oncological side-effects and associated costs, are important arguments to carefully consider the indication for blood transfusion. **Abbreviations BCa**: bladder cancer; CSS: cancer-specific survival; HR: hazard ratio; (N)MIBC: (non-) muscle-invasive BCa; OS: overall survival; PBT, perioperative blood transfusion; PRISMA, Preferred Reporting Items for Systematic Reviews and Meta-Analyses; RC: radical cystectomy; RFS: recurrence-free survival.

## Introduction

With ~549 393 newly diagnosed patients in 2018, bladder cancer (BCa) is the 11th most common cancer entity worldwide [1]. Radical cystectomy (RC) with pelvic lymphadenectomy is the current standard for the treatment of non-metastasised muscle-invasive BCa (MIBC) and highest-risk non-MIBC (NMIBC) [2]. Cancer-specific survival (CSS) and recurrence-free survival (RFS) in these patients varies greatly depending on pathological stage [3–5]. However, several perioperative risk factors for poor prognosis have been identified in recent years [6]. Perioperative blood transfusions (PBTs) were shown to be associated with worse survival outcomes in patients undergoing RC [7,8]. The impact of PBTs on oncological outcome has also been reported for other oncological entities such as lung cancer [9], hepatocellular carcinoma [10], and colorectal cancer [11]. The modulation of the immune system was proposed as a putative mechanism that might be responsible for a presumptive transfusion-related impact on prognosis [12]. Two previously published systematic reviews and meta-analyses assessed the impact of PBTs on BCa [13,14]. Wang *et al*. [14] and Cata *et al*. [13] conducted meta-analyses including six and eight studies with a total of 7080 and 15 655 patients in 2015 and 2016, respectively. Intriguingly, both found reduced overall survival (OS), CSS and RFS for patients receiving PBTs. Nevertheless, within the last few years, this topic has drawn increasing attention and there has been a rise in the number of new studies assessing the impact of PBT on oncological outcomes of patients with BCa. We therefore conducted a systematic review of new and previously published literature to provide up-to-date information on the impact of PBT on survival outcomes of patients undergoing RC.

## Methods

### Data sources

We searched PubMed, Ovid Medical Literature Analysis and Retrieval System Online (MEDLINE), Google Scholar, and the ClinicalTrials.gov databases from January 2010 to May 2020, with no limits of language or publication type. Used search terms were ‘cystectomy’ and ‘blood transfusion’, as well as ‘bladder cancer’ and ‘blood transfusion’ contained in the title or abstract. We also assessed the reference lists of relevant studies and previous reviews about this topic for additional studies. Assessment of possible sources was done according to the previously published Preferred Reporting Items for Systematic Reviews and Meta-Analyses (PRISMA) guidelines [15].

### Study selection

The main inclusion criteria included information on PBT (intra- and/or postoperative) in patients undergoing RC because of BCa and a statistical report on the prognostic impact. Blood transfusion could be allogenic, autologous or leucocyte-depleted. Studies had to report at least one of the following endpoints: OS, CSS and/or RFS. Randomised controlled trials, prospective cohorts, multicentre studies and retrospective studies were included. We excluded reviews, letters without original data, abstracts, poster presentations, and editorials. In the case of duplicate publications reported by the same author, either the higher quality or most recent publication was selected.

### Data extraction

Two investigators (Y.V. and G.B.S.) independently reviewed and extracted the information from each article, which met the inclusion criteria. Discussion and consensus resolved any potential disagreements. The main characteristics extracted were: (i) the authors name, (ii) publication year, (iii) country of origin, (iv) recruitment period, (v) median follow-up, (vi) study type, (vii) number of patients, (viii) transfusion rate (ix) median age, and (x) number of patients receiving neoadjuvant chemotherapy (NAC).

Extracted data regarding survival outcome (OS, CSS, and RFS) included the hazard ratios (HRs) with 95% CIs and the respective statistical *P* values. The HR was only reported if available from multivariable analyses. Univariable analyses were not reported. A *P* < 0.05 was considered to indicate statistical significance. Quality assessment of studies was done using the Newcastle–Ottawa Scale and studies with a minimum score of 6 were considered to be of ‘high quality’ [16].

## Results

### Characteristics

**Figure 1.**
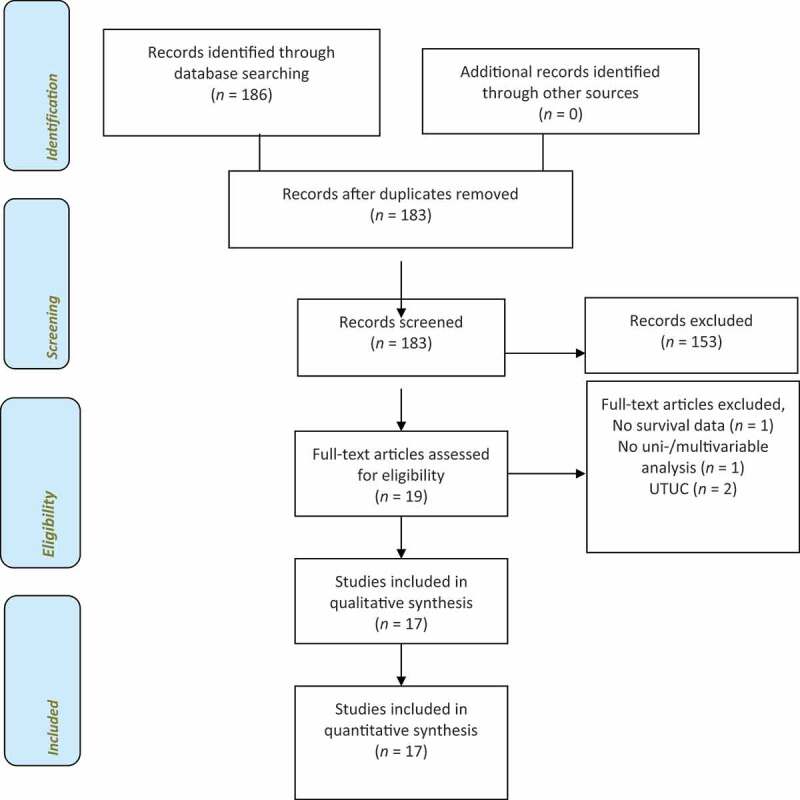
Flow diagram of study identification process

A total of 186 studies were identified and screened. After exclusion of duplicates, studies with insufficient data and studies not eligible regarding the topic, we identified 17 eligible studies that were assessed and included into this review (Figure 1). Overall, the studies included 19 627 patients. Key characteristics are shown in Table 1 [7,8,17–31]. All the studies had a retrospective design. Five studies assessed the timing of PBT and compared intra- and postoperative transfusions in multivariable analyses [7,17–20]. All other studies assessed transfusion perioperatively. The median follow-up time ranged from 7.8 [18] to 110 months [21]. The number of patients included in the studies ranged from 115 [18] to 4380 [22]. Transfusion rates ranged between 23.3% [23] and 72.9% [24].

**Table 1.** Characteristics of the included studies

Reference	Country	Recruitment period	Follow-up, months, median	Study type	No. of patients	Transfusion rate, *n* (%)	Time of transfusion, *n* (%)	Age, years, median	NAC, *n* (%)
Abel *et al*., 2014 [7]	USA	2003–2012	18.7	Retro.	360	241 (65.0)	Intraop. 66 (18) Postop. 79 (22) Intraop. and postop. 96 (27)	67.9	Excluded
Buchner *et al*., 2017 [17]	Germany	2004–2014	26.0	Retro.	722	317 (44.0)	Intraop. 263 (36) Postop. 132 (18) Intraop. and postop. 78 (11)	70	13 (1.8)
Chalfin *et al*., 2016 [18]	USA	2010–2013	7.8	Retro.	115	65 (56.5)	Intraop. 27 Postop. 40	N/A	115 (100.0)
Chipollini *et al*., 2016 [19]	USA	2008–2015	27.5	Retro.	1026	342 (33.2)	Intraop. 149 Postop. 58	68.8	387 (37.7)
Furrer et al., 2018 [23]	Switzerland	2000–2015	39.0	Retro.	885	187 (23.3)	N/A	N/A	137 (15.59)
Gierth *et al*., 2014 [20]	Germany	1995–2010	70.1	Retro.	350	219 (63.0)	Intraop. 183 (52) Postop. 99 (28)	68	0 (0.0)
Kluth *et al*., 2014 [28]	Multinational	1998–2010	36.1	Retro.	2895	1128 (39.0)	N/A	67	0 (0.0)
Lee *et al*., 2015 [24]	Korea	1991–2012	34/44	Retro.	432	315 (72.9)	N/A	N/A	47 (10.9)
Linder *et al*., 2013 [8]	USA	1980–2005	10.9	Retro.	2060	1279 (62.0)	N/A	69/66	115 (5.6)
Morgan *et al*., 2013 [29]	USA	2000–2008	25.0	Retro.	777	323 (41.6)	N/A	69.5	20 (2.6)
Moschini *et al*., 2016 [21]	Italy	1990–2013	110.0	Retro.	1490	580 (38.9)	N/A	68	43 (2.9)
Rosenblatt *et al*., 2020 [25]	Sweden	2008–2014	N/A	Retro.	120	40 (33.3)	N/A	69	120 (100.0)
Sadeghi *et al*., 2012 [26]	USA	1989–2010	25.5	Retro.	638	209 (32.8)	N/A	68.1 (mean)	N/A
Siemens *et al*., 2017 [30]	Canada	2000–2008	N/A	Retro.	2593	1608 (62.0)	N/A	N/A	Excluded
Soubra *et al*., 2015 [22]	USA	1992–2009	21.0	Retro.	4380	1139 (26.0)	N/A	74	N/A
Syan-Bhanvadia *et al*., 2017 [31]	USA	2010–2014	37.2	Retro.	173	46 (26.7)	N/A	70	59 (34.1)
Vetterlein *et al*., 2018 [27]	Multinational	2011	26.0	Retro.	611	315 (51.6)	N/A	68.1 (mean)	12 (2.0)

Intraop.: intraoperative; N/A, not available; postop.: postoperative; Retro.: retrospective.

### Overall survival (OS)

**Figure 2.**
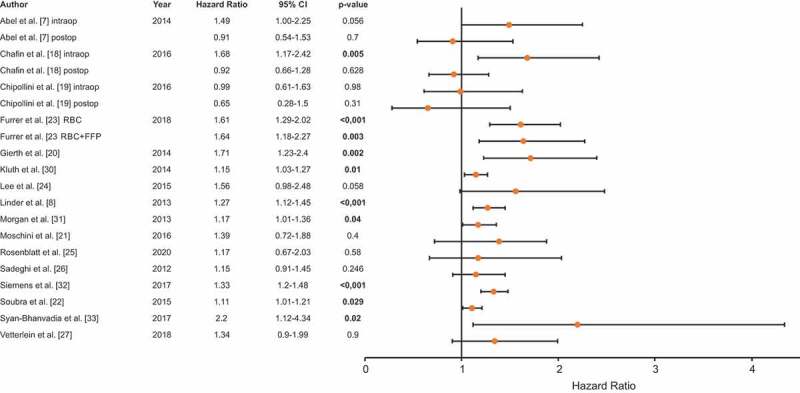
OS

The OS rate was reported by 16 studies, which included 18 905 patients. Nine studies reported a significant adverse impact of PBT on OS (Figure 2 [7,8,18–31]). Yet, seven studies reported no significant impact of blood transfusion on OS. Chalfin *et al*. [18] separately investigated intra- and postoperative PBT. Notably, intraoperative blood transfusion significantly predicted worse OS (HR 1.68, 95% CI 1.17–2.42; *P* = 0.005), whereas postoperative blood transfusions were not correlated with prognosis (HR 0.92, 95% CI 0.66–1.28; *P*= 0.628). Interestingly, both Abel *et al*. [7] and Chipollini *et al*. [19] reported no impact of PBT on OS for intra- or postoperative transfusions.

### Cancer-specific survival (CSS)

**Figure 3.**
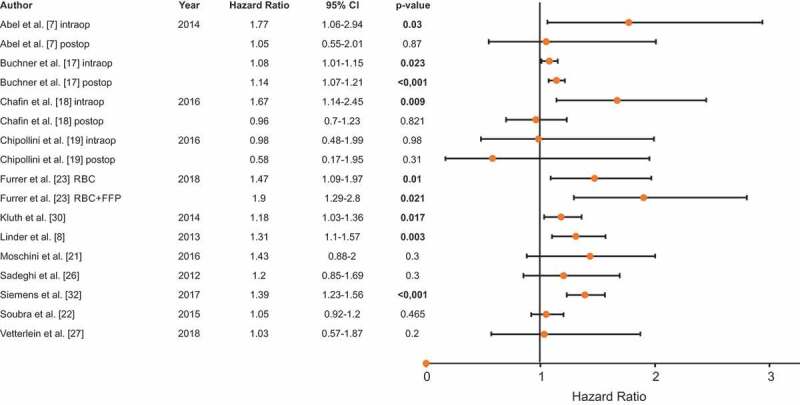
CSS

The CSS rate was reported by 12 studies, including 17 833 patients. Seven investigations reported a significantly reduced CSS in patients with PBT and the HRs ranged between 1.08 [17] and 1.90 [23] (Figure 3 [7,8,17–19,21-23,26–28,30]). The remaining five studies did not find any significant association between PBT and CSS. Abel *et al*. [7] and Chalfin *et al*. [18] reported a significantly lower cancer-specific prognosis only for intraoperative, but not postoperative blood transfusions. Conversely, Buchner *et al*. [17] reported an impact of intra- and postoperative blood transfusions on CSS (intraoperative: HR 1.08, 95% CI 1.01–1.15, *P* = 0.023; postoperative: HR 1.14, 95% CI 1.07–1.21, *P* < 0.001). Chipollini *et al*. [19] did not report any correlation of PBT with CSS, irrespective of the time point of transfusion.

### Recurrence-free survival (RFS)

**Figure 4.**
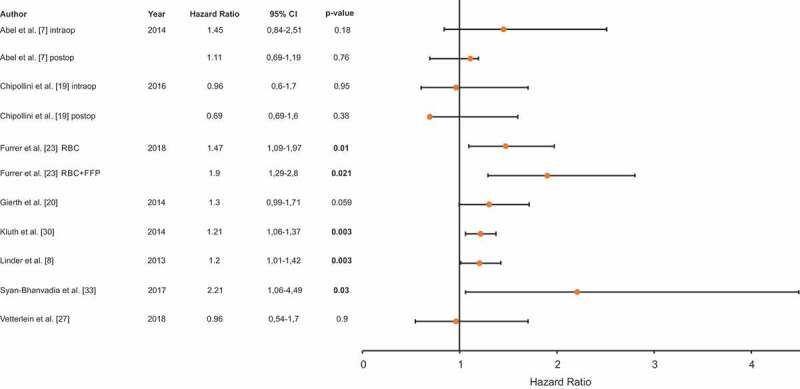
RFS

Eight studies assessed the impact of PBT on BCa recurrence, including a total number of 8245 patients (Figure 4 [7,8,19,20,23,27,28,31]). There were four studies reporting significantly worse RFS in patients treated with PBT. Conversely, four studies found no significant impact of PBT on recurrence of BCa after RC. The two studies separately investigating intra- and postoperative transfusions did not show any correlation between PBT and RFS [7,19].

## Discussion

Our present systematic review included a total of 17 studies compared to six and eight studies included in the previous analyses.

The majority of studies correlated PBT with significantly worse OS. However, seven of nine studies reported no independent impact of PBT on OS [7,19,24–27,32]. Notably, four of the six studies including >1000 patients were able to demonstrate a significant correlation between PBT and worse OS, lending support to the importance of avoiding unnecessary PBT. To date, two meta-analyses have analysed the association of PBT with prognosis in patients with BCa [13,14]. In their meta-analyses published in 2015 and 2016, both, Cata *et al*. [13] and Wang *et al*. [14] concluded that PBT was associated with worse OS, CSS and RFS in patients undergoing RC for BCa. However, the number of new studies concerning this topic has risen since then. With ~85 million blood transfusions given to patients each year worldwide, this intervention remains one of our most common clinical practices [33]. Several studies were able to demonstrate impaired survival outcomes for different cancer entities in patients receiving PBT [7,8,17,20,22,23,28–31]. Different independent retrospective studies analysed the impact of PBT on oncological outcomes of patients with BCa. Proposed mechanisms that may explain the reduced survival include the induction of immunosuppressive effects by PBT [34,35], which was given the term ‘transfusion-related immunomodulation’ (TRIM) [36]. Cell-derived microparticles from blood cells might play a key role in transfusion-related effects and the infusion of growth factors, such as vascular endothelial growth factor and transforming growth factor β, could also lead to a stimulation of cancer cell proliferation [37,38]. Colvin *et al*. [39] even proposed anaesthetic agents to lead to an immune function impairment and therefore cause cancer cells to spread. Yet, the surgery itself may lead to reduced host immunity due to tissue injury and may therefore be a mediator of the effect of PBT on survival outcomes [40].

Interestingly, Chalfin *et al*. [18] were able to show that the timing of blood transfusion was associated with worse OS. That study found a significantly worse OS rate if patients received a blood transfusion intraoperatively. Conversely, there was no impact on OS if the blood transfusion was given postoperatively. As the underlying biology is still poorly understood, it seems to be premature to speculate on explanations for this observation.

The majority of studies that analysed CSS showed a significantly reduced CSS. Importantly, the currently largest study, including 4380 patients, also reported a significantly reduced CSS. However, five of the 12 studies reporting on CSS were not able to correlate PBT with prognosis. Similarly to OS, two studies showed that timing of PBT is relevant when correlating PBT and CSS [7,18].

The data regarding RFS are  rather inconclusive, as half of the studies reported a significantly worse RFS and the other half did not show any impact of PBT on RFS.

Interestingly, several other factors may also lead to a different impact of PBT and survival. Moschini *et al*. [41] were able to show that only intraoperative blood transfusions caused a significant impact on cancer-specific mortality and overall mortality, yet they did not find any difference in ABO blood type. Furthermore, the role of preoperative anaemia seems to play a role in mediating the detrimental effects of PBT. However, the recent literature seems to be contradictive. Moschini *et al*. [21] found PBT to have especially detrimental effects on survival outcomes if the patient was not anaemic before RC. On the contrary, Gierth *et al*. [42] found preoperative anaemia itself to worsen survival outcomes. Several studies investigated the impact of intra- and postoperative transfusions separately. However, as the results were heterogeneous and the definition of the postoperative period varied significantly, it is too early for any conclusions.

As RC is a semi-elective surgery, it might be reasonable to therapeutically target preoperative anaemia. However, a recently published randomised trial investigating preoperative iron supplementation failed to reduce blood transfusions [43].

Collectively, our present systematic review shows a presumptive adverse impact of PBT on the prognosis of patients with BCa undergoing RC, which is consistent with previously published meta-analyses. However, several obstacles impede a conclusion on this important question. First of all, all studies published to date have had a retrospective design. Secondly, there was no clear definition of PBTs. Thirdly, the cohorts were heterogeneous regarding tumour stage, frailty, adjuvant therapy, and NAC.

As the underlying mechanism behind transfusion-related biological processes is still poorly understood, future studies should address this question. Additionally, it would be interesting to see if blood transfusions during NAC before RC impede prognosis.

## Conclusion

PBT was associated with worse survival outcomes in the majority of the studies. As all investigations were of a retrospective design and a number of studies reported no correlation between PBT and prognosis, the level of evidence is limited. Translational studies investigating the underlying biological mechanism(s) might help to improve blood transfusion management.
